# A Rare Case of Acute Pancreatitis in the Setting of Hemorrhagic Cholecystitis

**DOI:** 10.7759/cureus.22546

**Published:** 2022-02-23

**Authors:** Chelsea R Yap, Ruchir Puri

**Affiliations:** 1 General Surgery, University of Florida College of Medicine – Jacksonville, Jacksonville, USA

**Keywords:** hemobilia, biliary pancreatitis, hemorrhagic cholecystitis, blood clots, anticoagulation

## Abstract

Hemorrhagic cholecystitis is a rare form of acute cholecystitis with very few cases reported in the literature. We report a novel case of a 79-year-old male who developed hemorrhagic cholecystitis and concomitant acute pancreatitis. The patient presented to the emergency department with a one-day history of severe epigastric pain radiating to his back. The patient was on an anticoagulant therapy for a history of pulmonary embolism. He had an elevated serum lipase and on a right upper quadrant ultrasound, a mildly distended gallbladder without stones was noted. Computed tomography (CT) the following day demonstrated heterogenous material in the gallbladder concerning for blood clots. Laparoscopic cholecystectomy with intraoperative cholangiogram (IOC) was performed that revealed a gallbladder filled with clots. He had an uneventful post-operative recovery.

## Introduction

Hemorrhagic cholecystitis is an uncommon presentation of acute cholecystitis. Acute pancreatitis in association with it has not been reported previously. Hemorrhage into the gallbladder lumen can occur for a variety of reasons including trauma, obstructive cholecystitis, percutaneous interventions, biliary neoplasms, biliary parasites, or bleeding disorders [1]. Imaging modalities such as ultrasound (US) and computed tomography (CT) can help make the diagnosis by demonstrating characteristic findings of a distended gallbladder filled with heterogenous materials and a thickened gallbladder wall [1]. Here, we report a case of a patient on chronic anticoagulation for a history of pulmonary embolism who presented with signs and symptoms of acute pancreatitis and was ultimately found to have hemorrhagic cholecystitis.

## Case presentation

A 79-year-old male presented to the emergency department with a one-day history of severe epigastric pain radiating to his back with associated nausea and vomiting. He denied any fevers, chills, or changes in his bowel habits. He was on 5 mg of apixaban, twice a day, after developing a pulmonary embolism after a right total hip arthroplasty in March 2018. Additional past medical history was significant for hypertension, diabetes mellitus, Parkinson’s disease, coronary artery disease (status post coronary artery bypass graft) and he was a former smoker. He denied any alcohol intake. On physical exam, he was extremely tender in both his right upper quadrant (RUQ) and epigastric region with a positive Murphy’s sign. His white cell count was 11.49 x 10^3^/uL, hemoglobin was 15.4 g/dL and lipase was >3000 U/L. Liver function tests (LFTs) revealed a total bilirubin of 0.9 mg/dL and aspartate aminotransferase (AST) and alanine aminotransferase (ALT) were within normal limits. His prothrombin time (PT) was 14.7 seconds and international normalized ratio (INR) was 1.3. Right upper quadrant ultrasound did not show cholelithiasis or cholecystitis but did show a distended gallbladder. His apixaban was held, and he was placed on a heparin drip in anticipation of any interventions during his hospital stay.

**Figure 1:**
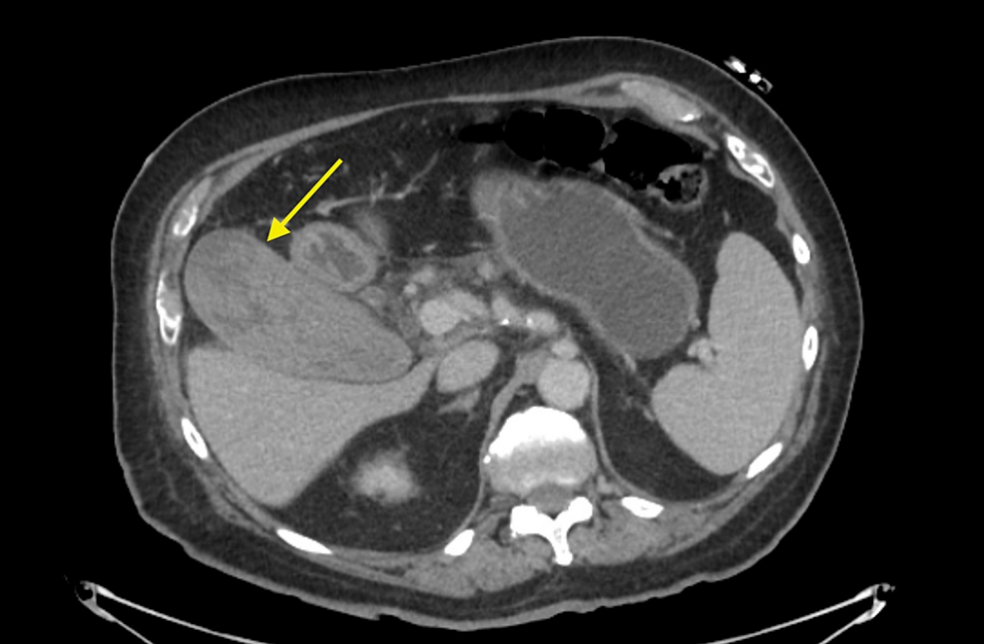
Preoperative CT pancreas protocol demonstrating a distended gallbladder with mixed hyperdensities.

**Figure 2:**
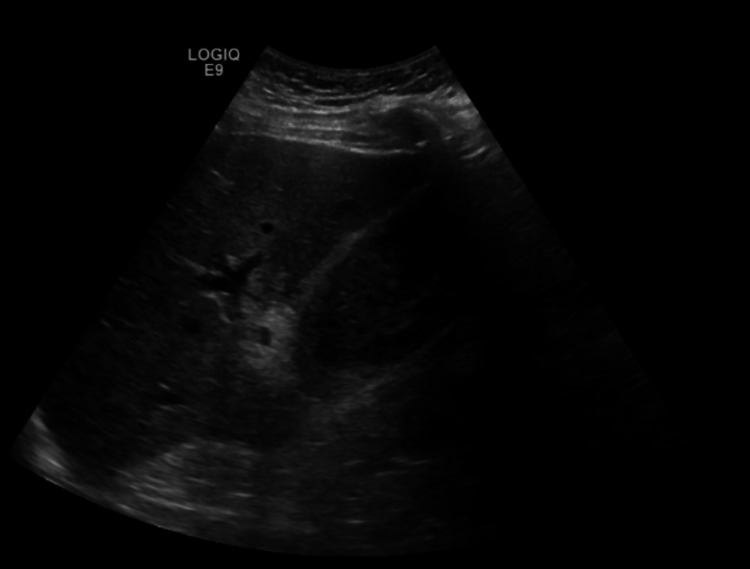
Repeat RUQ US redemonstrating distended gallbladder filled with echogenic material and a now thickened gallbladder wall measuring 0.4 cm. RUQ: right upper quadrant

On hospital day 2, total bilirubin increased to 4.2 mg/dL with a direct component of 3.7 mg/dL and his hemoglobin decreased to 13.2 g/dL. AST and ALT increased to 673 IU/L and 84 IU/L respectively, alkaline phosphatase was noted to be 161 IU/L (Table 1). A CT pancreas protocol was obtained to evaluate the patient’s known pancreatic uncinate process mass that measured 1.6 cm and was unchanged since the prior exam. It also re-demonstrated a distended gallbladder, however, now with mixed hyperdense material and dilation of common bile duct up to 1 cm (Figure 1). A repeat right upper quadrant ultrasound was performed on hospital day 3, and again, the gallbladder was distended but now filled with echogenic material and a now thickened gallbladder wall measuring 0.4 cm (Figure 2). The bilirubin decreased to 1.5 mg/dL the next day, which leads us to believe that the patient either had transient hemobilia or passed some clots from the gallbladder to the common bile duct.

**Table 1:** Laboratory trends Dbili: direct bilirubin; Tbili: total bilirubin; AST: aspartate aminotransferase; ALT: alanine aminotransferase; ALP: alkaline phosphatase.

	Day 1	Day 2	Day 3	Reference Range
Dbili	0.4	3.7	0.7	0.0-0.2 mg/dL
Tbili	0.9	4.2	1.5	0.2-1.0 mg/dL
AST	22	673	248	14-33 IU/L
ALT	13	84	122	10-42 IU/L
ALP	70	161	158	40-129 IU/L

**Figure 3:**
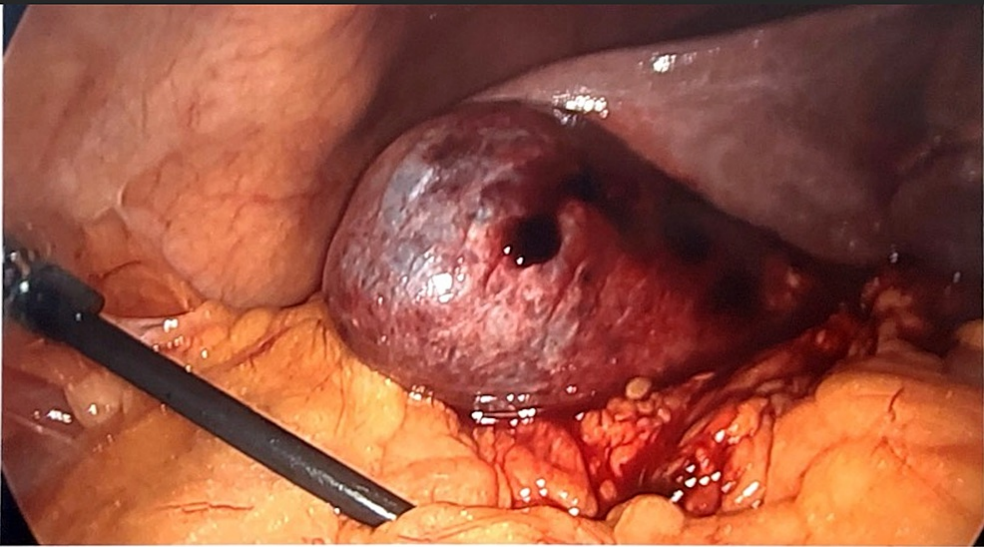
Gallbladder is distended and the wall appears congested and bruised

**Figure 4:**
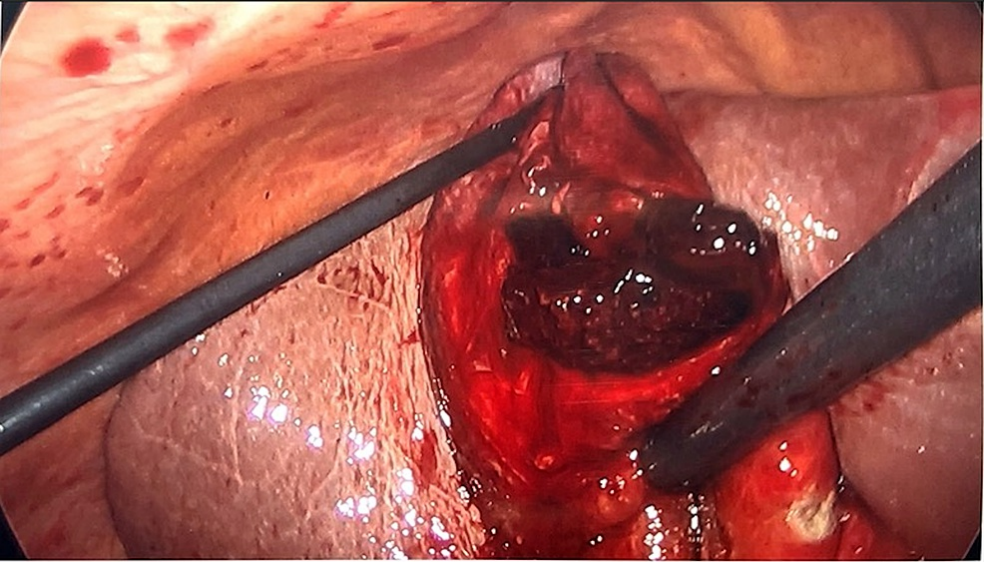
Clot being evacuated from the gallbladder

**Figure 5:**
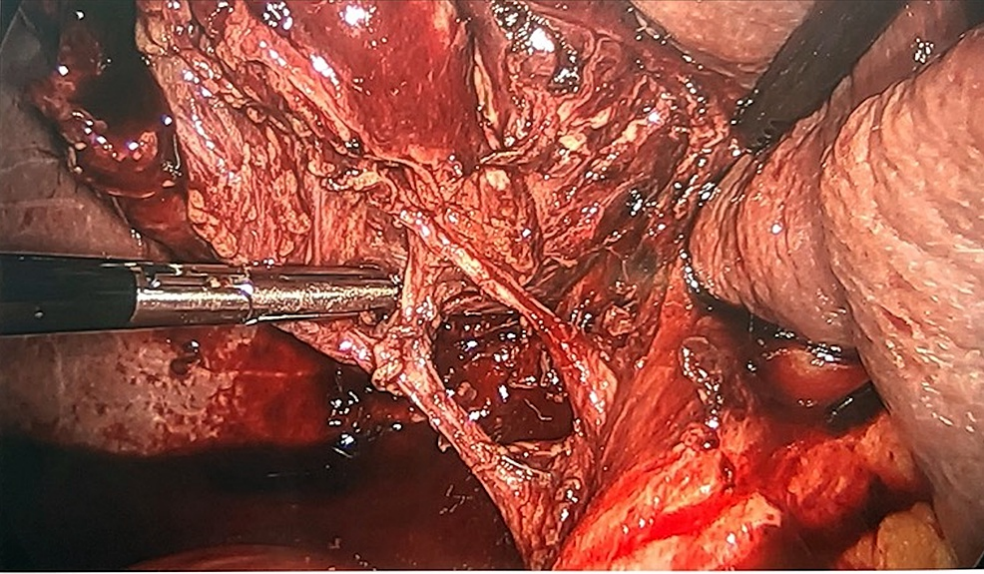
Critical view of safety showing the cystic artery and cystic duct

**Figure 6:**
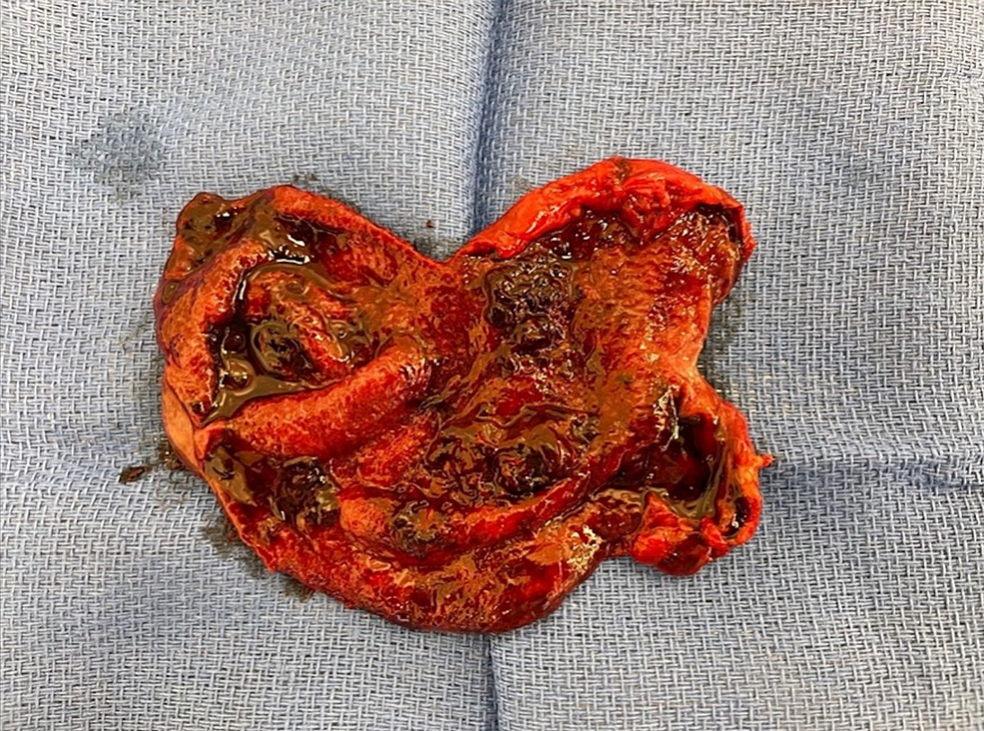
Gross specimen showing intraluminal clot inside of the gallbladder

Given the concern for hemorrhagic cholecystitis and likely biliary pancreatitis caused by hemobilia or blood clots, he consented for a laparoscopic cholecystectomy and intraoperative cholangiogram (IOC). The gallbladder was grossly distended and needle decompression was attempted but unsuccessful due to large clots (Figure 3). We opened the gallbladder with cautery and old blood and clots were evacuated (Figure 4). The cystic duct and artery were skeletonized completely and the critical view of safety was obtained (Figure 5). Before performing the IOC, the cystic duct was milked and a few small clots were evacuated. IOC revealed no filling defects in the biliary tree. Morphologic examination of the gallbladder showed hemorrhagic cholecystitis with clots but without stones (Figure 6). Postoperative recovery was uneventful, he was restarted on a heparin drip and transitioned to apixaban over the next 48 hours.

## Discussion

Hemorrhagic cholecystitis was first described in the literature in 1979 by Shah and Clegg [2]. Hemorrhage into the gallbladder lumen is a rare complication of acute cholecystitis that could potentially be fatal if not recognized and treated early [3]. Hemorrhagic cholecystitis can occur as a result of iatrogenic and non-iatrogenic causes. It has been reported to occur in the setting of trauma, malignancy, and bleeding diathesis, such as renal failure, cirrhosis, and anticoagulation [4]. It can be difficult to make the diagnosis of hemorrhagic cholecystitis, as it mimics other disorders, but there were 30 case reports between 1985 and 2018, of which the majority were managed with cholecystectomy [5].

The mechanism by which hemorrhagic cholecystitis occurs is thought to be due to a transmural inflammatory process that causes erosion of the mucosa and ischemia which then leads to further mucosal breakdown and subsequent erosion into gallbladder vessels. This can then lead to either hemorrhage into the gallbladder lumen or the abdominal cavity [6]. Another theory is that cholelithiasis could cause microbleeding and small ulcerations from the gallbladder which would normally heal on its own, however, in the setting of a bleeding disorder or anticoagulation therapy, such is not the case [7]. Of note, our patient did not have cholelithiasis.

There are several types or presentations described in the literature for patients with hemorrhagic cholecystitis, one of which is the blood staying clotted in the gallbladder resulting in distension and ultimately perforation into the abdomen causing peritonitis. In addition, the blood may enter the bowel lumen and patients can present with hematemesis or melena. Finally, blood clots can travel to the common bile duct and result in a presentation similar to choledocholithiasis [6]. There has not been any other reported case of acute pancreatitis in the setting of hemorrhagic cholecystitis.

The physical and laboratory findings in a patient with hemorrhagic cholecystitis are very similar to that of calculous acute cholecystitis, and it is crucial to obtain a thorough history and inquire if patients are on anticoagulation or have liver or kidney disease that could predispose them to bleeding [7]. Imaging can demonstrate characteristic findings on ultrasound, such as a distended gallbladder with thickened wall and heterogenous materials. The heterogenous echogenic material is blood in different stages of clotting and is typically non-shadowing, non-mobile [8]. CT findings are essentially the same as those on an US, however, the arterial phase of a contrast-enhanced CT can identify active extravasation of contrast into the gallbladder if present [9].

Laparoscopic cholecystectomy is the gold standard for the management of benign biliary diseases such as stones, polyps, and cholecystitis and is also the treatment of choice for hemorrhagic cholecystitis [3]. Cholecystostomy should be reserved for those patients who are not stable enough for the operating room and would not tolerate surgical intervention, but it is conceivable that clots would not be successfully evacuated by a drain alone.

Given the widespread utilization of anticoagulation therapy, hemorrhagic cholecystitis should be considered in the differential diagnosis of patients that present with signs and symptoms of acute cholecystitis in the absence of stones or sludge. In our patient, we are unsure why the LFTs were normal at presentation. We speculate that he had started bleeding and passing small clots prior to the presentation that led to pancreatitis and over the course of the admission developed full-blown hemorrhagic cholecystitis.

Alcoholic and biliary pancreatitis are well-known etiologies of pancreatitis, other common causes include gallstones, hypertriglyceridemia, hypercalcemia, pancreas divisum, and medications. At his initial presentation, we assumed that he had biliary pancreatitis since he denied a history of alcohol intake. The patient's triglycerides and calcium were within normal limits. After the finding of hemorrhagic cholecystitis intraoperatively, a workup for pancreas divisum was not performed. As mentioned, his admission US was normal but once we obtained the repeat US and CT scan that showed hyperdense material in the gallbladder, we started entertaining additional etiologies of cholecystitis. Differential diagnosis of hyperdense material in the gallbladder includes extensive sludge, gallbladder polyposis, adenomyomatosis, and gallbladder carcinoma [10]. Our primary concern was gallbladder carcinoma, but since his preoperative US was normal and there were no signs of invasive disease on the CT scan, we thought this was less likely. In addition, given the relatively acute change in the US during the admission, we favored bleeding and clots in the gallbladder given his history of anticoagulation. Our findings were confirmed intraoperatively. As mentioned above, we suspect that hemobilia or passage of small clots may have precipitated the pancreatitis. Hemobilia is sometimes characterized by Quincke’s triad, an association of right upper quadrant pain, jaundice, and gastrointestinal bleeding (GIB) [11]. Our patient did not have the typical presentation of hemobilia as he was not jaundiced and did not have gastrointestinal bleeding despite extensive clot burden in the gallbladder.

## Conclusions

Hemorrhagic cholecystitis is an uncommon manifestation of acute cholecystitis, and concomitant acute pancreatitis is even rarer. The diagnosis should be considered in patients who are on anticoagulation that present with sign and symptoms of acute cholecystitis but without obvious gall stones or sludge. Laparoscopic cholecystectomy is well tolerated and results in effective removal of the clot burden.
